# Sex- and estrous-cycle dependent dorsal hippocampal phosphoproteomic changes induced by low-dose ketamine

**DOI:** 10.1038/s41598-022-05937-x

**Published:** 2022-02-02

**Authors:** Samantha K. Saland, Kathrin Wilczak, Edward Voss, TuKiet T. Lam, Mohamed Kabbaj

**Affiliations:** 1 Department of Biomedical Sciences, College of Medicine, Florida State University, 1115 W Call Street, Tallahassee, FL 32306 USA; 2 Program in Neuroscience, College of Medicine, Florida State University, 1115 W Call Street, Tallahassee, FL 32306 USA; 3 Keck MD & Proteomics Resource, Yale School of Medicine, Yale University, New Haven, CT USA; 4 Department of Molecular Biophysics and Biochemistry, Yale University, New Haven, CT USA

**Keywords:** Neuroscience, Proteomic analysis

## Abstract

Numerous emotional and cognitive processes mediated by the hippocampus present differences between sexes and can be markedly influenced by hormonal status in males and females of several species. In rodents, the dorsal hippocampus (dHPC) is known to contribute to the rapid antidepressant actions of the NMDA receptor antagonist ketamine. We and others have demonstrated a greater sensitivity to the fast-acting antidepressant ketamine in female versus male rats that is estrogen- and progesterone-dependent. However, the underlying mechanisms remain unclear. Using an acute low dose (2.5 mg/kg) of ketamine that is behaviorally effective in female but not male rats, a label-free phosphoproteomics approach was employed to identify ketamine-induced changes in signaling pathway activation and phosphoprotein abundance within the dHPC of intact adult male rats and female rats in either diestrus or proestrus. At baseline, males and females showed striking dissimilarities in the dHPC proteome and phosphoproteome related to synaptic signaling and mitochondrial function—differences also strongly influenced by cycle stage in female rats. Notably, phosphoproteins enriched in PKA signaling emerged as being both significantly sex-dependent at baseline and also the primary target of ketamine-induced protein phosphorylation selectively in female rats, regardless of cycle stage. Reduced phosphoprotein abundance within this pathway was observed in males, suggesting bi-directional effects of low-dose ketamine between sexes. These findings present biological sex and hormonal milieu as critical modulators of ketamine’s rapid actions within this brain region and provide greater insight into potential translational and post-translational processes underlying sex- and hormone-dependent modulation of ketamine’s therapeutic effects.

## Introduction

Males and females across several species differ at baseline in a number of higher-order emotional^1,2^ and cognitive^3,4^ processes, whose disturbance also vary in severity as a function of sex in disease or disease-like states^5^. Among the various brain regions subserving such functions, the hippocampus not only plays critical roles in learning and memory^6,7^, but is also implicated in neuropsychiatric disorders such as depression which exhibit sex differences in prevalence, symptom expression and treatment response^8^. Despite the existing body of literature, there still remains an incomplete picture of how hippocampal structure and function differ—or not—between males and females, and how this may contribute to normal and disrupted cognitive and emotional processes in either sex. Structurally, clinical studies have identified both larger^9^ and smaller^10^, as well as no difference^11,12^, in total hippocampal volumes in males versus females. However, when accounting for differences in total hippocampal volume, subregion-specific sex differences within the hippocampus emerge^13^, highlighting the importance of factoring in region specificity when examining this structure.

Cognitive dysfunction has long been considered a prominent and enduring feature of depression^14,15^. In particular, hippocampus-dependent memory impairment has been identified as one of the most significant measures of cognitive dysfunction observed in depressed patients when examined across several domains^16^. Animal models of chronic stress also produce impairments in spatial memory mediated by the dorsal hippocampus (dHPC) in mice^17^. Given observations of reduced hippocampal volume in patients with mood disorders^18,19^, spatial memory may be of particular interest as a target for antidepressant treatment^15^. With equivocal effectiveness of traditional antidepressant treatments on cognitive impairment in depressed individuals^20^, these deficits are enduring and can persist independently of other mood symptoms^21^. Excitingly, a single infusion of the rapid-acting antidepressant ketamine was recently found to improve episodic memory impairment in patients with treatment-resistant depression acutely and for up to 7 days following treatment^22^, and can be sustained with repeated treatment^23,24^.

Given its involvement in both cognitive and emotion-relevant functions, the dHPC in particular represents an interesting convergence point for investigation of potential sex differences at baseline, as well as sex-specific response to fast-acting antidepressant medications that may affect outcomes in both of these behavioral arenas. To this end, the dHPC has been implicated in ketamine’s rapid antidepressant-like effects in rodents. One predominant mechanism here suggested to contribute to the fast-acting behavioral response to low-dose ketamine involves reduction of eEF2 kinase (eEF2K) activity and subsequent dephosphorylation of eEF2 via suppression of spontaneous NMDA receptor activity. This cascade of events results in disinhibition of elongation in protein translation and a rapid translation-dependent increase in dHPC BDNF protein levels^25^. Previous work from our lab^26–29^ and others^30^ has repeatedly demonstrated enhanced antidepressant-like and pro-hedonic behavioral sensitivity of female rodents to low-dose ketamine at baseline and under acutely stressful situations, accompanied by sex-dependent molecular correlates of these behavioral effects within the medial prefrontal cortex (mPFC) and dHPC^26,27^. Whereas male rats exhibit increased mPFC synaptoneurosomal mTOR phosphorylation as well as decreased p-eEF2 in the dHPC in response to 5 mg/kg ketamine, neither of these effects can be observed in female rats following a lower, behaviorally-effective dose of ketamine (2.5 mg/kg) in this sex, suggesting neither of these well-established mediators of ketamine’s rapid antidepressant-like effects mediate heightened female behavioral sensitivity to this drug^26^. Although rapid de-suppression of translation via inhibition of eEF2K activity was not observed in females 30 min post-treatment, we subsequently identified sex-dependent elevations in dHPC BDNF protein levels 24 h after 2.5 mg/kg ketamine associated with pro-hedonic effects selectively in female rats^27^, suggesting the possibility of sex-dependent dHPC involvement in the time course of ketamine’s behavioral actions.

While pharmacokinetic differences may contribute to differential behavioral sensitivity to ketamine in male and female rodents^30,31^, relative levels of gonadal hormones in female rats do not appear to impact metabolism or distribution of low-dose ketamine in plasma or within the dHPC^31^. With this in mind, we have previously shown that enhanced female responsivity to ketamine’s rapid antidepressant-like effects is dependent upon gonadal estradiol (E2) and progesterone (P4) in rats^26,27^ and varies with the stage of estrous cycle during which ketamine is administered in mice^28^, suggesting that this sex-dependent behavioral sensitivity may be modulated by relative levels of circulating gonadal hormones in freely cycling intact rodents. This discrepancy between the absence of hormonal influence on pharmacokinetics in female rodents and presence of their effects on behavioral sensitivity to ketamine—at least at very low doses—may be resolved by pharmacodynamic sex and estrous cycle differences at baseline, in response to ketamine, or both. However, the contribution of baseline sex differences in protein abundance and phosphorylation to low-dose ketamine’s effects within behaviorally-relevant brain regions such as the dHPC remains unclear—as does the extent to which circulating levels of E2 and P4 in females may contribute in an activational manner to rapid ketamine-induced changes in phosphorylation patterns.

To this point, an understanding of baseline sex differences in the dHPC proteome and phosphoproteome is currently lacking, and would provide a foundation upon which to examine rapid drug induced changes in this brain region and their modulation by sex and or/circulating hormone levels. Importantly, apart from sex- or hormone-dependent differences in behavioral response to a drug, similar behavioral outcomes between males and females may also arise through distinct molecular processes^32^. Therefore, here we sought to explore baseline sex differences in the dHPC proteome and phosphoproteome at baseline and in response to ketamine to determine if—and to what extent—such baseline differences may predict rapid alterations in signaling pathway activation by ketamine at a low dose behaviorally-relevant selectively in female rats. In addition, given the necessity of ovarian-derived hormones for the heightened female sensitivity to low-dose ketamine, female rats in either diestrus (low E2/P4) or proestrus (high E2/P4) stages of their estrous cycle were examined to identify potential hormone-dependent changes in protein phosphorylation patterns within this sex.

## Materials and methods

### Animals

Adult male (250–270 g) and female (200–225 g) Sprague–Dawley rats (Charles River, Wilmington, MA) were pair-housed in 43 × 21.5 × 25.5 cm plastic cages and maintained on a 12 h:12 h light:dark cycle (lights on at 0700) in a temperature- and humidity-controlled room. Food and water were available ad libitum throughout the duration of the study. All animal protocols were carried out in accordance with the NIH Guide for Care and Use of Laboratory Animals and recommendations in the ARRIVE guidelines, and were approved by the Institutional Animal Care and Use Committee of Florida State University.

### Handling and estrous cycle monitoring

Male and female rats were habituated to basic handling procedures daily for 1 week upon arrival. Following habituation, daily estrous cycle monitoring and stage assignment of intact female rats was performed via vaginal lavage and characterization of cytologic smears as previously detailed^27,31,33^. Post-mortem smears were also collected to confirm staging assignments. Only rats exhibiting at least two consecutive 4-day cycles were used in the present work. Three female rats not meeting this criterion were excluded prior to group assignment and analysis. To minimize potential handling and stress confounds between sexes, male rats received a similar brief daily handling in parallel.

### Ketamine treatment and tissue collection

**Figure 1:**
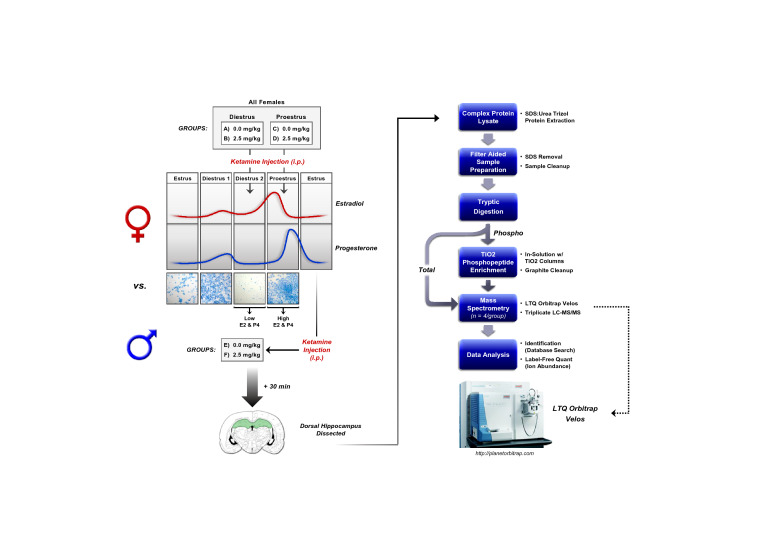
Phosphoproteomics experimental design and sample preparation workflow. *i.p.* intraperitoneal, *SDS* sodium dodecyl sulfate, *TiO2* titanium dioxide, *LC–MS/MS* liquid chromatography-tandem mass spectrometry.

The complete experimental design including sample preparation and analysis workflow are presented in Fig. 1. Separate adult male (n = 4/group) and female rats in either proestrus (high E2, P4; n = 4/group) or diestrus (low E2, P4; n = 4/group) received a single intraperitoneal (*i.p.*) injection of either saline (SAL) or 2.5 mg/kg racemic ketamine hydrochloride (KET; Butler Schein Animal Health, Inc., Dublin, OH) and were sacrificed 30 min later under non-stressful conditions. Brains were rapidly removed, snap-frozen in 2-methylbutane, and stored at − 80 °C until further processing for mass spectrometric analysis. Saline and KET were administered at a volume of 1 mL/kg between 1400 and 1500 h. This range provides an approximate timeframe when levels of circulating E2 and P4 are elevated in proestrus or low in diestrus female rats^34^, and was chosen in accordance with our previous work^26,27,31^ to determine whether relative levels of these hormones in females at the time of KET administration influence rapid protein phosphorylation and signal transduction.

### Sample preparation and tryptic digestion

Tissue punches (1.0–2.0 mm) were taken bilaterally from 200 µm sections of the dorsal hippocampus (dHPC), taking care to avoid inclusion of surrounding cortical and striatal structures. Total protein was then extracted using TRI-Reagent (Molecular Research Center, Cincinnati, OH) according to the manufacturer’s protocol as previously described^27,35,36^. Protein resuspended in 1:1 (v/v) 1% SDS:8 M Urea was stored at − 80 °C until further processing. Filter Aided Sample Preparation (FASP) was performed according to an established protocol^37^ with some modifications to remove salt and detergent from samples prior to tryptic digestion and phosphopeptide enrichment. Here, 30 µL or 60 µL supernatant (250 µg protein max) for total or phosphoprotein fractions, respectively, was added to an Amicon Ultra-0.5 10 K centrifugal filter unit (UCF501024; Millipore, Billerica, MA) and washed twice with 200 µL UA buffer (8 M urea in 100 mM tris–HCl, pH 8.5) via centrifugation for 40 min at 14,000×*g* (20 °C). Samples were then incubated in the dark for 30 min with 50 mM iodoacetamide in UA buffer, spun for at 14,000 × *g* (20 °C) for 30 min and washed twice with 100 µL 50 mM ammonium bicarbonate. Filter units were carefully inverted into new collection tubes and centrifuged for 2 min at 1000×*g* to collect protein extract, followed by quantification of protein concentration by optical density spectroscopy using an ND-1000 Spectrophotometer (Nanodrop Technologies, Wilmington, DE). Fifty or 200 µg recovered protein for total or phosphoprotein fractions, respectively, were digested overnight at 37 °C with sequencing grade modified trypsin at 1 µg trypsin per 50 µg protein. Complex and phosphopeptide-enriched protein digests were dried via SpeedVac and submitted to the Florida State University Translational Science Laboratory for LC–MS/MS analysis (liquid chromatography (LC)-tandem mass spectrometry (MS/MS).

### TiO_2_ phosphopeptide enrichment and graphite clean-up

Enrichment of phosphopeptides from complex protein digests was performed using the Pierce TiO_2_ Phosphopeptide Enrichment and Clean-up Kit (Pierce Biotechnology, Rockford, IL) according to the manufacturer’s protocol. Briefly, TiO_2_ spin tips were first preconditioned with 20 µL Buffer A (0.4% trifluoroacetic acid (TFA)/80% acetonitrile (ACN)) and 20 µL Buffer B (25.7% lactic acid/71.4% Buffer A). Phosphopeptide binding was achieved by applying 200 µg of dried protein digest resuspended in 150 µL of Buffer B to spin tips and centrifuging for 10 min at 1000×*g*. Sample was reapplied and the process repeated, followed by one Buffer B wash and three with Buffer A. Spin tips were transferred into new collection tubes for recovery of phosphopeptides via centrifugation at 1000×*g*—first with 50 µL Elution Buffer 1 (5% ammonium hydroxide (NH_4_OH)), then 50 µL Elution Buffer 2 (5% pyrrolidine). Recovered phosphopeptide-enriched fractions were acidified with 100 µL 2.5% TFA to ensure proper binding for subsequent clean-up steps. Samples were processed with Pierce Graphite Spin Columns (Pierce Biotechnology, Rockfold, IL) according to manufacturer’s protocol to desalt prior to LC–MS/MS.

### LC–MS/MS

The LC–MS/MS method and parameters used were similar to those previously described^38^. Briefly, an externally calibrated Thermo LTQ Orbitrap Velos (High Resolution Electrospray Tandem Mass Spectrometer) equipped with a 2 cm, 100 µm i.d. (internal diameter) trap column (SC001 Easy Column; Thermo Scientific, Waltham, MA) followed by a 10 cm analytical column of 75 µm i.d. (SC200 Easy Column; Thermo Scientific, Waltham, MA) was used for LC–MS/MS. Both the trap column and analytical column contained C18-AQ packaging. The separation was carried out using Easy nano LCII (Thermo Scientific, Waltham, MA) with a continuous vented column configuration. A 5 µL (~ 500 ng) sample was taken into a 20 µL sample loop and loaded onto the trap. A flow rate of 300 nL/min was used for separation on the analytical column. Mobile phase A was composed of 99.9% H_2_O (EMD Omni Solvent) and 0.1% formic acid while mobile phase B was composed of 99.9% ACN and 0. 1% formic acid. A 1 h linear gradient from 0 to 45% B was executed. The LC eluent was nano sprayed directly into an LTQ Orbitrap Velos mass spectrometer. The LTQ Orbitrap Velos was set in a data-dependent mode and under direct control of the Xcalibur software (Thermo Scientific, Waltham, MA). The MS data was acquired using the parameters, 10 data-dependent collisional induced dissociation (CID) MS/MS scans per full scan (400 to 2000 m/z) at a mass resolution for MS1 of 60,000. The minimum signal required to trigger MS2 was 500, MS mass range 0 to 1,000,000, and dynamic exclusion enabled with following parameters: Repeat count: 1, Repeat Duration: 30.00 s, exclusion list size: 500, exclusion duration: 60.00 s and exclusion mass width relative to low and high mass: 10 ppm. All measurements were conducted at room temperature, and three technical replicates for each of the four biological replicates per treatment condition were run to enable statistical comparisons between samples.

### Label-free data analysis

A label-free quantitation approach using Progenesis QI (Nonlinear Dynamics, LLC, UK; version 2.0) similar to that found in Bordner, et al. was used to obtain quantitative information on peptides and proteins^39^. Here, the LC–MS/MS data (uninterpreted MS/MS spectra) were processed with protein identification carried out using an in-house Mascot search engine (version 2.4^40^). The complex and TiO2-enriched phosphopeptide fractions were analyzed separately. Resulting quantitative analyses at the protein level for the complex and phosphopeptide-enriched fractions were first combined and analyzed as a set to gain a global picture of differentially abundant proteins and phosphoproteins influenced in the dHPC by sex and/or estrous cycle. Subsequently, complex (or total) and phosphopeptide-enriched fractions were analyzed independently at the protein level for each comparison set. The Progenesis QI software performs chromatographic/spectral alignment (wherein one run is chosen as a reference to which all other data files are aligned), mass spectral peak picking and filtering, and quantitation of proteins and peptides. A normalization factor for each run was calculated to account for differences in sample load between injections as well as differences in ionization. The normalization factor was determined by calculating a quantitative abundance ratio between the reference run and the run being normalized, with the assumption being that most proteins/peptides are not changing in the experiment so the quantitative value should equal 1. The experimental design was setup to group multiple injections (technical and biological replicates) from each run into each comparison sets. No exclusions were made prior to analysis. The algorithm then calculated the tabulated raw and normalized abundances and ANOVA p-values for each feature in the data set. The MS/MS spectra were exported in .mgf (Mascot generic files) format for database searching. The Mascot search algorithm was used for searching against the Swiss Protein database with taxonomy restricted to *Rattus Norvegicus.* Carbamidomethyl (Cys), oxidation of Met, Phospho (Ser, Thr, Tyr), and Carbamyl (N-term) were entered as variable modifications. Two missed tryptic cleavages were allowed, precursor mass tolerance was set to 20 ppm, and fragment mass tolerance was set to 0.6 Da. The significance threshold was set based on a False Discovery Rate (FDR) of 1%. The Mascot search results were exported as .xml files and then imported into the processed dataset in Progenesis QI software where peptides identified were synced with the corresponding quantified spectral features. Protein abundances were then calculated from the sum of all unique normalized non-conflicting peptide ions for a specific protein on each run.

### Enrichment analyses

Pathway and functional analyses of protein and phosphoprotein abundance significantly differentially regulated by sex, estrous cycle and low-dose ketamine treatment were performed with Ingenuity Pathway Analysis (IPA) software (QIAGEN, Inc., https://www.qiagenbioinformatics.com/products/ingenuity-pathway-analysis)^41^. To further aid in this interpretation, significantly differentially abundant proteins and phosphoproteins were annotated with gene ontology (GO) terms, and overrepresentation analyses were carried out using the PANTHER term enrichment services^42^. Here, Fisher’s Exact test was used to determine overrepresentation, with a false discovery rate (FDR) correction cutoff set at *p* < 0.05. GraphPad Prism 9.0 software (GraphPad Software Inc., San Diego, CA) was used for production of volcano plots and GO figures.

## Results and discussion

### Sex-dependent total and phosphoprotein expression in the dHPC

**Figure 2:**
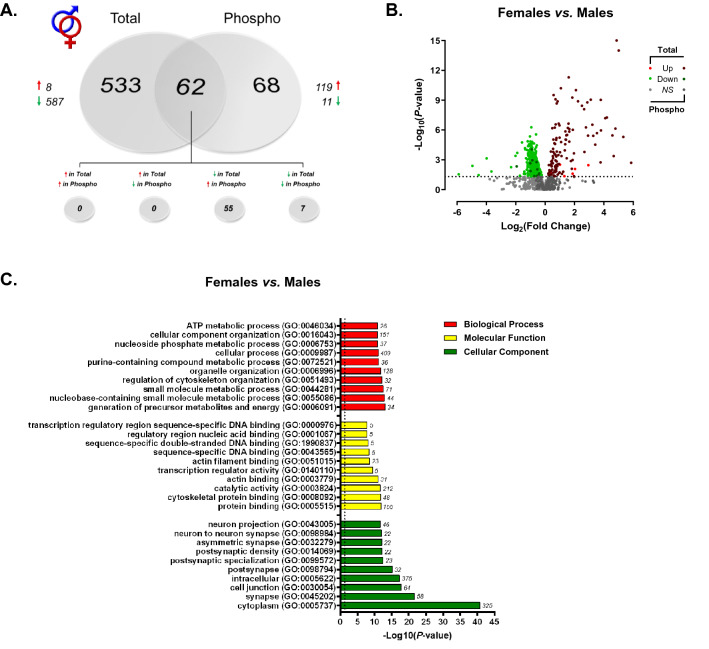
Sex-dependent total and phosphoprotein expression in the dHPC. (**A**) Venn diagram showing significantly lower total protein abundance, but greater phosphoprotein abundance in the dHPC of female *vs.* male rats. Each circle represents the significant female vs. male differences in abundance of proteins (“Total”) or phosphoproteins (“Phospho”), with the overlap representing proteins whose levels differed in both fractions. (**B**) Volcano plot depicting significant differences in protein and phosphoprotein abundance between female and male rats. Log_2_(Fold Change) values > 0 (red) indicate higher, and those < 0 (green) lower, abundance in females compared to males. Dots above the horizontal dashed line represent proteins with *p* < 0.05 (FDR threshold set at 1%). (**C**) Top 10 most significant (*p* < 0.05) gene ontology (GO) enrichment terms per category of proteins and phosphoproteins with significant sex-dependent abundance differences.

Using a label-free shotgun approach, we provide here the first characterization of the rat dorsal hippocampal proteome and phosphoproteome across sex and estrous cycle. When analyzing global differences in the dorsal hippocampal proteome of male and female rats, a total of 7310 features mapping to 1027 unique proteins were confidently identified and submitted for label-free quantitation. Raw and normalized ion abundance, confidence scores, and statistical analyses of all identified proteins and features can be found in Supplementary Tables S1 and S2, respectively. Of those 1027 quantified proteins, 595 were found to be significantly differentially abundant between sexes (Fig. 2A)—a majority (587) of which displayed lower abundance in the female dorsal hippocampus compared to that of males. A similar trend in greater male hippocampal protein abundance was recently reported in the synaptic proteome of adult mice^43^; however, conflicting evidence has also been reported in the rat^44^ and mouse^45^ which identified either greater female-to-male protein abundance or no difference, respectively. However, these studies were performed using the entire hippocampus (dorsal to ventral), rather than a selective analysis of the dorsal portion examined herein. As well, methodological differences in type of tissue (organotypic slice culture, as in^44^), method of collection and age of subjects are likely to contribute to this discrepancy.

While differences in total protein levels can provide insight into potential functional differences mediated by such proteins at the cellular level, these data are limited in their ability to indicate protein activity levels and therefore their contribution to sex-dependent signal transduction and pathway modulation. To address this, we implemented a parallel examination of the global phosphoproteome via TiO2-enriched fractions from the same lysate of each biological replicate to provide a more comprehensive picture of overall activity across a variety of signaling pathways in the dHPC of both sexes. Between males and females, 1193 peptides representing 280 unique proteins were quantified from phosphopeptide-enriched fractions of hippocampal lysate (Supplementary Tables S3, S4). Location of phosphorylation sites were identified for 984/1193 quantified peptides and are presented in Supplementary Table S4. Interestingly, among the 130 phosphoproteins whose levels significantly differed between male and female rats, 119 were greater and 11 lower in abundance in female versus male rats (Fig. 2A). The volcano plot in Fig. 2B illustrates the notable contrast between the significant differences in total dHPC protein vs phosphoprotein abundance between male and female rats, where females exhibited greater phosphorylation but lower levels of unmodified protein. Of the 130 differentially abundant phosphoproteins, 62 shared different levels of both the unmodified and phosphorylated protein—52 with greater phosphoprotein but lower total protein abundance in female compared to male rats, 7 with lower levels of both total and phosphorylated protein forms. Given that females exhibited greater levels of phosphorylation than males but lower abundance of several unmodified proteins, it is unclear what the effect of greater female phosphoprotein abundance here is on the net throughput of the signaling pathways within which these proteins participate.

When considering proteins and phosphoproteins differentially abundant between males and females as a whole (i.e., total and TiO2-enriched fractions analyzed together), GO analyses (Fig. 2C) revealed overrepresentation of intracellular proteins localized to the cytoplasm, but more significantly, the synapse—glutamatergic synapses, in particular (GO: 0098978, *p* = 0.00217). Molecular function and biological process GO terms enriched in actin/cytoskeletal organization, ATP metabolic processes and energy generation, as well as transcription regulator activity were generally observed (Fig. 2C). Taken together, ontology enrichment across these 3 primary domains appears to indicate that the major sex-dependent differences in the dHPC proteome and phosphoproteome globally affect structural protein dynamics, ATP metabolic processes/energy metabolism and enzymatic activity at the level of the synapse and mitochondria.

**Figure 3:**
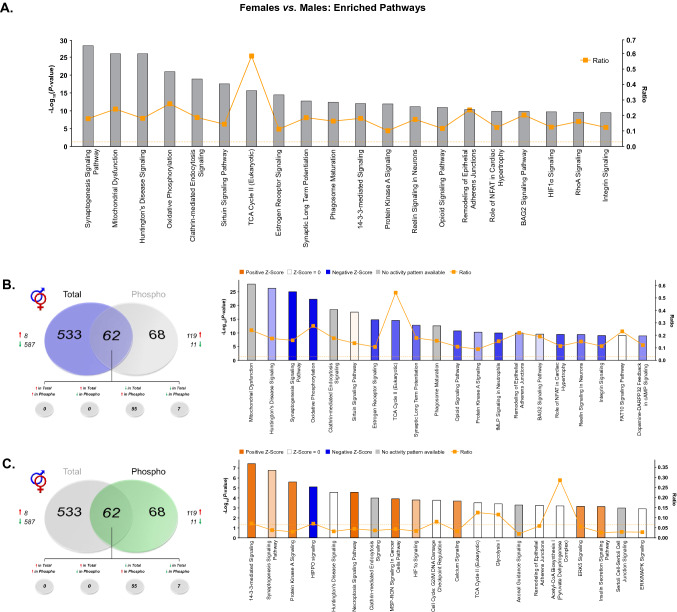
IPA enrichment analyses of differentially-abundant proteins and phosphoproteins between female and male rats. (**A**) Canonical pathway enrichment for combined list of proteins and phosphoproteins significantly differentially abundant between female and male rats. Separate IPA analyses of total (**B**) and phosphoproteins (**C**) with sex-dependent abundance describe contributions of each fraction to predicted activity levels within enriched pathways. Based upon the direction of fold change of significant differentially abundant proteins/phosphoproteins within the dataset, the *Z*-Score serves as a predictor of pathway activation states by assessing the match between the observed and predicted (evidence-based) up/down regulation patterns (see^41^). Pathways are colored by predicted activation state: orange (activated) or blue (inhibited), where darker colors represent higher absolute *Z*-scores. Z-scores for pathways colored in gray could not be calculated due to insufficient evidence for a prediction of either activation or inhibition to be made. The ratio (solid orange line) represents the number of molecules within the comparison dataset meeting the cutoff criterion (*p* < 0.05; Fisher’s Exact Test), divided by the number of molecules comprising a given pathway within the reference set (Ingenuity Knowledge Base). Bar graphs were produced in Ingenuity Pathway Analysis (IPA) software (QIAGEN, Inc.).

In order to further understand the contribution of these sex differences to various signaling processes, Ingenuity Pathway Analysis (IPA) was first performed on the combined list of significantly differentially abundant proteins and phosphoproteins to obtain a global picture of pathway activity differences within the dHPC of male and female rats (Fig. 3A, Supplementary Table S5). Here, the sex-dependent abundance of numerous proteins (phosphorylated or not) enriched in synaptogenesis and synaptic long-term potentiation agrees with ontological overrepresentation of proteins localized to synapse-associated cellular compartments. As well, lower overall expression of proteins involved in the mitochondrial dysfunction pathway were observed in the female dHPC when compared to males. Total and phosphopeptide-enriched fractions were next analyzed independently to determine whether differentially-abundant phosphoproteins were more selectively targeted to certain dHPC signaling pathways affected globally by sex. Indeed, whereas mitochondrial function enrichment was more restricted to total proteins exhibiting differential abundance between sexes (Fig. 3B), phosphoproteins whose abundance was significantly greater in the female dHPC were primarily related to synaptogenesis and protein kinase A (PKA) signaling pathways (Fig. 3C). Of note, related mitochondria-localized signaling pathways were found to be significantly enriched in both total and phosphoprotein fractions. Here, females exhibited lower predicted activity within the TCA Cycle II pathway relative to males at the total protein level, in addition to sex-biased enrichment of the Acetyl-CoA Biosynthesis signaling pathway in phosphoprotein-enriched fractions. The glucose metabolite acetyl-CoA is a substrate for the TCA cycle, which upon entering the mitochondrial matrix may be converted to glutamate through a number of intermediary steps^46^. That dHPC protein abundance within the TCA signaling pathway was lower in female compared to male rats may reflect baseline differences in energy metabolism and glutamate cycling within this brain region. These findings align with previously-observed elevations in TCA cycle protein abundance in male versus female rats following proteomic analysis of organotypic hippocampal cultures^44^. Interestingly, altered glutamate cycling has been shown in the hippocampus of depressed patients^47^. Likewise, male rats exposed to chronic stress exhibit reductions of hippocampal TCA cycle proteins^48^. Given the baseline sex differences observed in TCA cycle signaling, and that hippocampal energy metabolism has been shown to contribute to the antidepressant-like effects of ketamine in male mice^49^, it is possible that this pathway may be a functionally relevant target of low-dose ketamine in females.

### Estrous cycle-dependent total and phosphoprotein expression in the dHPC of female rats

#### Widespread effects of estrous cycle on the female dHPC proteome

**Figure 4:**
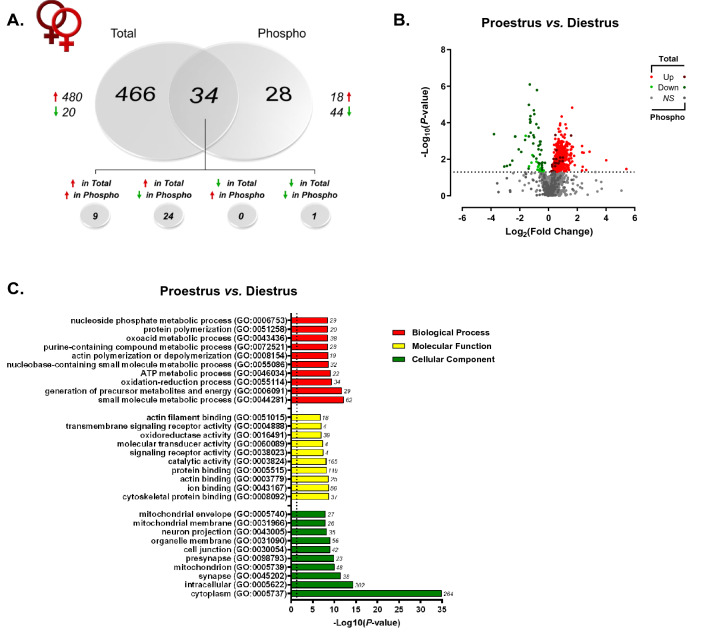
Estrous cycle-dependent total and phosphoprotein expression in the dHPC. (**A**) Venn diagram showing number of total (“Total”) and phosphoproteins (“Phospho”) with significant differential abundance between female rats in either proestrus or diestrus. Red arrows indicate greater levels observed in proestrus *vs.* diestrus females. (**B**) Volcano plot depicting significant differences in protein and phosphoprotein abundance between proestrus and diestrus female rats. Log_2_(Fold Change) values > 0 (red) indicate higher, and those < 0 (green) lower, abundance in proestrus females compared to diestrus females. Dots above the horizontal dashed line represent proteins with *p* < 0.05 (FDR threshold set at 1%). (**C**) Top 10 most significant (*p* < 0.05) gene ontology (GO) enrichment terms per category of proteins and phosphoproteins with significant estrous cycle-dependent abundance differences.

Given the known influence of ovarian-derived hormones on dHPC-mediated behaviors in rodents^50,51^, within-sex protein and phosphoprotein levels were next compared between proestrus and diestrus female rats. Here, we identified a total of 6944 features mapping to 960 unique proteins which were then submitted for label-free quantitation. Raw and normalized ion abundance, confidence scores, and statistical analyses of all identified proteins and peptides can be found in Supplementary Tables S6 and S7, respectively. Interestingly, of those proteins quantified, 500 were found to be significantly differentially abundant between proestrus (high E2P4) and diestrus (low E2P4) female rats (Fig. 4A)—96% of which were observed at greater levels in proestrus as compared to diestrus female rats. In contrast, while only 62 of the 274 quantified phosphoproteins (representing 1227 peptides; Supplementary Tables S8 and S9) differed by estrous cycle stage, their abundance was significantly lower in proestrus compared to diestrus females—44 of those 62 differentially abundant were significantly lower in the former group of females, 24 of which were detected at greater levels for the unmodified protein. The volcano plot in Fig. 4B more clearly illustrates these findings. Therefore, while estrous cycle stage did appear to impact both the dHPC proteome and phosphoproteome, the magnitude of this hormone-dependent impact was much greater on total versus phosphorylated protein levels. This is consistent with reports of significant estrous cycle-dependent fluctuations in protein and transcript levels within this brain region^43,44,52–54^ and others such as the medial prefrontal cortex^55^.

**Figure 5:**
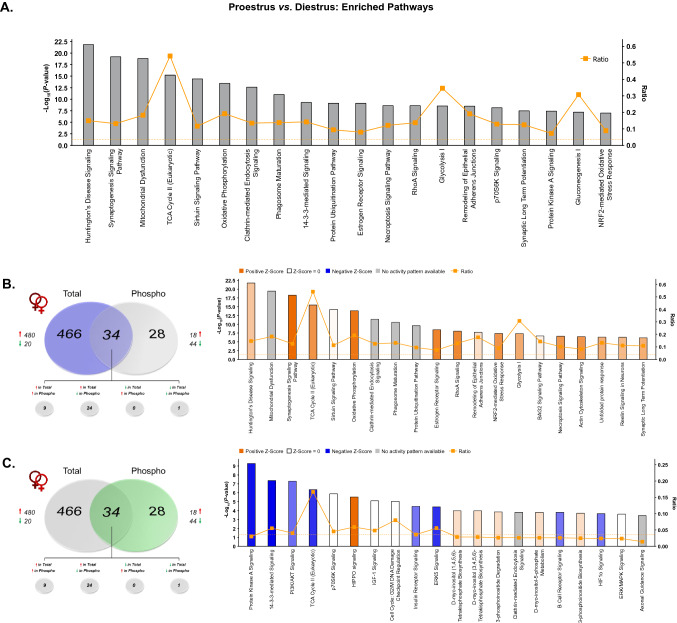
IPA enrichment analyses of proteins and phosphoproteins differentially abundant between proestrus and diestrus female rats. (**A**) Canonical pathway enrichment for combined list of proteins and phosphoproteins significantly differentially abundant between proestrus and diestrus female rats. Separate IPA analyses of total (**B**) and phosphoproteins (**C**) with estrous cycle-dependent abundance describe contributions of each fraction to predicted activity levels within enriched pathways. Based upon the direction of fold change of significant differentially abundant proteins/phosphoproteins within the dataset, the *Z*-Score serves as a predictor of pathway activation states by assessing the match between the observed and predicted (evidence-based) up/down regulation patterns^41^. Pathways are colored by predicted activation state: orange (activated) or blue (inhibited), where darker colors represent higher absolute *Z*-scores. Z-scores for pathways colored in gray could not be calculated due to insufficient evidence for a prediction of either activation or inhibition to be made. The ratio (solid orange line) represents the number of molecules within the comparison dataset meeting the cutoff criterion (*p* < 0.05; Fisher’s Exact Test), divided by the number of molecules comprising a given pathway within the reference set (Ingenuity Knowledge Base). Bar graphs were produced in Ingenuity Pathway Analysis (IPA) software (QIAGEN, Inc.).

When total and TiO2-enriched fractions analyzed together, GO analyses (Fig. 4C) identified significant overrepresentation of cycle-dependent proteins located in synapse- and mitochondrial-associated cellular compartments, similar to what was observed between sexes. Accordingly, biological processes and molecular functions related to signaling receptor activity/transmembrane signaling and energy metabolism represented a significant majority of the hormone-sensitive changes in protein and phosphoprotein abundance. IPA analysis of this same combined protein set (Fig. 5A; Supplementary Table S10) representing global estrous cycle-dependent changes in the proteome (Fig. 5B) and phosphoproteome (Fig. 5C) further confirmed findings from GO analyses. Specifically, 3 of the most significantly-enriched canonical pathways were synaptogenesis signaling, mitochondrial dysfunction and TCA cycle II signaling—thus confirming the importance of cyclical hormone-mediated changes to both the dHPC synaptic protein machinery^52,53^, as well as to mitochondrial energy metabolism processes which—while up to this point not yet identified in the dHPC—have been shown elsewhere as being heavily influenced by estrous cycle in the mPFC^55^.

#### Differential contribution of estrous cycle stage to sex differences in the dHPC proteome and phosphoproteome

**Figure 6:**
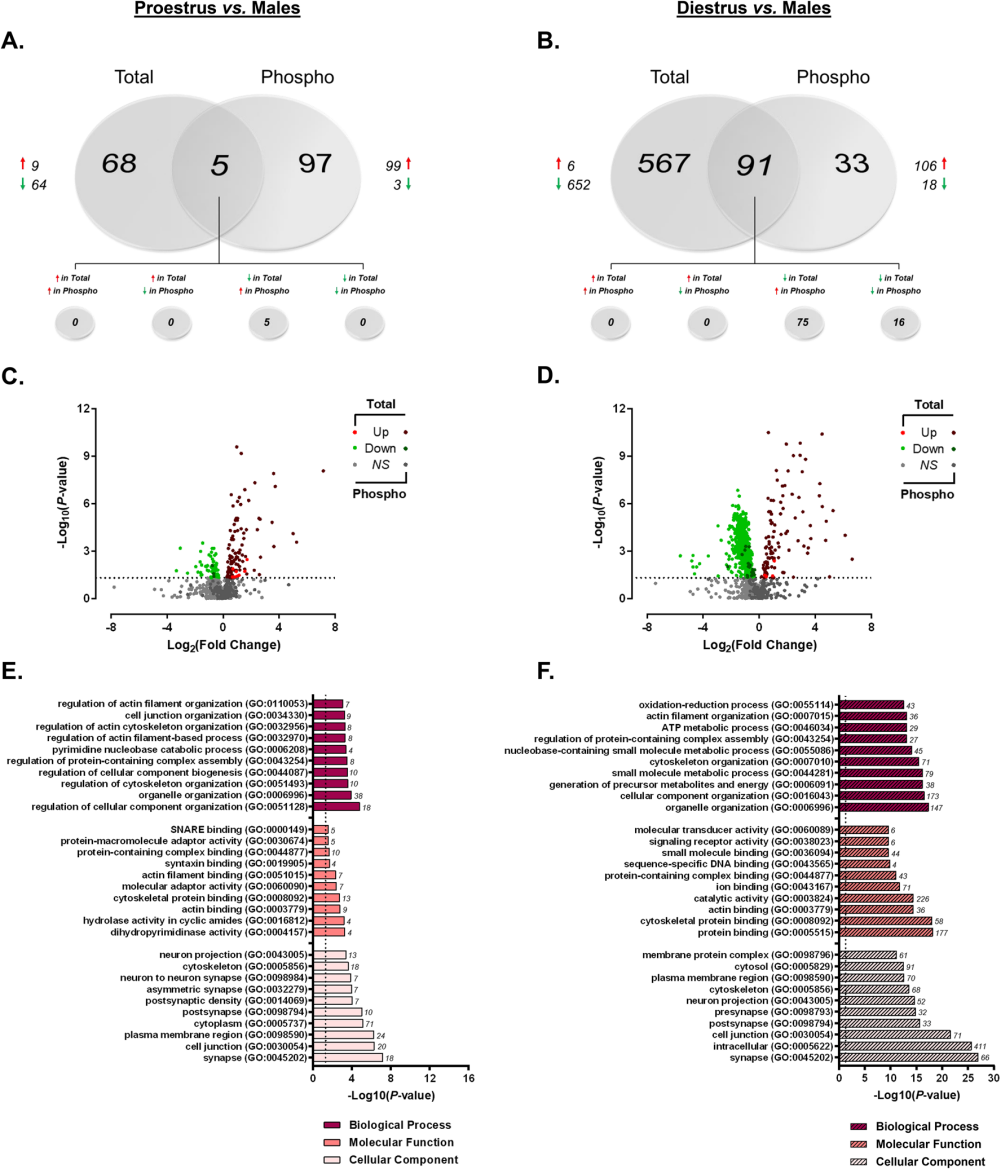
Estrous cycle influences sex bias in dHPC proteome and phosphoproteome. (**A**,**B**) Venn diagrams representing the number of differentially-abundant total- (“Total”) and phosphoproteins (“Phospho”) between males and females in either proestrus (**A**) or diestrus (**B**). Red arrows indicate greater abundance observed in proestrus/diestrus females *vs.* males. (**C**,**D**) Volcano plots depicting significant differences in protein and phosphoprotein levels between proestrus (**C**) or diestrus (**D**) females and males. Log_2_(Fold Change) values > 0 (red) indicate higher, and those < 0 (green) lower, abundance in proestrus/diestrus females compared to males. Dots above the horizontal dashed line represent proteins with *p* < 0.05 (FDR threshold set at 1%). (**E**,**F**) Top 10 most significant (*p* < 0.05) gene ontology (GO) enrichment terms per category of proteins and phosphoproteins with significant estrous cycle-specific sex bias in abundance between proestrus (**E**) or diestrus (**F**) female rats and male rats.

With the substantial estrous cycle-dependent differences in protein abundance observed between proestrus and diestrus female rats, we next sought to determine the extent to which the estrous cycle might contribute to the differential protein and phosphoprotein abundance observed between sexes. As presented in Fig. 6, the contribution of estrous cycle to the vast dHPC differences observed between female and male rats was markedly apparent (Fig. 6A–D). When comparing proestrus females to males (Fig. 6A), 982 unique proteins comprised of 8998 peptides were identified (Supplementary Tables S11 and S12). Of these quantified proteins, 73 were found to be significantly different in abundance between proestrus females and males—a majority (64/73) of which were lower in females. From TiO2-enriched fractions, we identified 1027 peptides and 257 proteins which were submitted for quantitation (Supplementary Tables S13 and S14). Here, 102 phosphoproteins were differentially abundant between proestrus females and male rats. As previously observed when grouped females were compared with males, nearly all (97%) of these phosphoproteins were present in significantly higher levels in proestrus females. The same trend was also observed when comparing of the phosphoproteome of diestrus females to male rats (Fig. 6B), where 106 of the 124 differentially abundant phosphoproteins were greater in the diestrus female rats (Supplementary Tables S15 and S16). The clearest estrous cycle-dependent differences between sexes were observed when comparing total protein levels for proestrus and diestrus females independently against those in males. When comparing diestrus females to males, we identified a total of 7251 peptides and 958 unmodified proteins, 652 of which were present at significantly lower levels in diestrus versus males (Supplementary Tables S17 and S18). Only 6 proteins were greater in abundance in the dHPC of this group of females (Fig. 6B). The volcano plots for proestrus (Fig. 6C) and diestrus (Fig. 6D) females versus males comparisons illustrate the distinct female bias in greater phosphoprotein abundance (independent of estrous cycle stage), while also making apparent the estrous cycle-dependence of the predominantly reduced total protein levels described above when comparing grouped females against males.

In order to further differentiate the functional attributes of proteins and phosphoproteins exhibiting estrous cycle-dependent sex bias, GO overrepresentation analyses were performed separately for proestrus (Fig. 6E) and diestrus (Fig. 6F) female versus male comparisons. Assessment of the cellular locations within which these differentially-abundant proteins act revealed common overrepresentation within synaptic (GO:0045202) and cytoskeletal (GO:0005856) compartments between proestrus and diestrus rats when each group was independently compared to males. Apart from a common enrichment in general protein and cytoskeletal/actin binding, the overrepresented molecular functions and biological processes diverge for proteins and phosphoproteins whose levels differ from males depending on estrous cycle stage. When comparing proestrus female rats to male rats (Fig. 6E), biological processes for differentially abundant proteins and phosphoproteins were predominantly related to organelle, cytoskeletal and cell junction organization. Functionally, many of these proteins are generally implicated in protein binding—particularly cytoskeletal/actin and SNARE complex binding. Taken together, the proteins and phosphoproteins differentially abundant between proestrus females and males predict functional differences in molecular adaptor activity involving structural organization and vesicle docking machinery at the level of the synapse.

Apart from the commonly-regulated cytoskeletal protein binding, differentially abundant proteins and phosphoproteins in diestrus females compared to males were enriched in signal transduction-related molecular functions. In addition, biological processes involved in derivation of metabolic energy for ATP generation were significantly overrepresented in this combined subset of differentially-abundant proteins and phosphoproteins (Fig. 6F). These data suggest diestrus-selective sex differences in proteins participating in the regulation of physiological neuronal function that are highly localized at synaptic compartments; whereas dHPC proteins and phosphoproteins in proestrus females differ from males more so in processes involved in vesicle fusion-associated protein binding and activity at the synapse. Commonly regulated proteins exhibiting sex differences, independent of estrous cycle, are cytoskeletal binding and organizational proteins and phosphoproteins.

**Figure 7:**
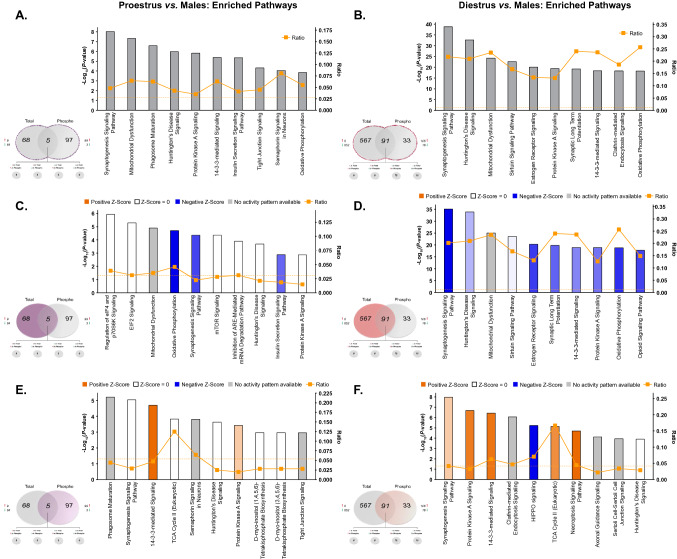
IPA enrichment analyses of estrous cycle-specific differences in protein and phosphoprotein abundance between female and male rats. (**A**,**B**) Canonical pathway enrichment for combined list of proteins and phosphoproteins identified as differentially abundant (*p* < 0.05) from proestrus *vs.* males (**A**) and diestrus *vs.* males comparisons. Separate IPA analyses of total proteins (**C**,**D**) and phosphoproteins (**E**,**F**) with estrous cycle-specific sex differences in abundance describe contributions of each fraction to predicted activity levels within enriched pathways. Based upon the direction of fold change of significant differentially abundant proteins/phosphoproteins within the dataset, the *Z*-Score serves as a predictor of pathway activation states by assessing the match between the observed and predicted (evidence-based) up/down regulation patterns^41^. Pathways are colored by predicted activation state: orange (activated) or blue (inhibited), where darker colors represent higher absolute *Z*-scores. Z-scores for pathways colored in gray could not be calculated due to insufficient evidence for a prediction of either activation or inhibition to be made. The ratio (solid orange line) represents the number of molecules within the comparison dataset meeting the cutoff criterion (*p* < 0.05; Fisher’s Exact Test), divided by the number of molecules comprising a given pathway within the reference set (Ingenuity Knowledge Base). Bar graphs were produced in Ingenuity Pathway Analysis (IPA) software (QIAGEN, Inc.).

Ingenuity pathway analyses were next performed to uncover canonical pathways commonly and differentially affected by estrous cycle stage in female rats compared to males. When lists of significantly differentially-abundant proteins and phosphoproteins were combined, the top 10 pathways enriched between proestrus and diestrus versus male comparisons were remarkably similar (Fig. 7A,B; Supplementary Tables S19 and S20). Among the most commonly-affected pathways between female and male rats—regardless of estrous cycle stage—were synaptogenesis signaling, mitochondrial dysfunction, PKA signaling and oxidative phosphorylation. This is unsurprising, as the same pathways were among the most significantly affected by sex when grouped females were compared to males (Fig. 3)—the important point of emphasis here being that independent analysis between females and males stratified by estrous cycle confirms probable sex bias within these pathways, rather than hormone status-dependence. However, given the differential contribution of total versus phosphoproteins to sex differences in signaling pathways identified in the present work (Fig. 3), pathway enrichment analyses were performed separately for significantly differentially-abundant total and phosphoproteins to visualize how estrous cycle may predict sex differences in phosphorylation status of proteins independently of total protein levels.

Here, individual analysis of differentially-abundant proteins of males compared to either proestrus (Fig. 7C) or diestrus females (Fig. 7D) revealed that the commonly-affected pathways present in both comparisons were primarily represented by lower levels of unmodified proteins in female groups compared to males. These estrous cycle-independent differences resulted in predicted reduced activity within synaptogenesis signaling and oxidative phosphorylation pathways. However, the same total protein analyses also uncovered divergent estrous cycle-specific sex differences in pathway activity. In particular, dHPC proteins whose levels significantly differed between proestrus females and males were uniquely involved in protein synthesis-related pathways—these include regulation of eIF4 p70S6K and eIF2 signaling, mTOR signaling and inhibition of ARE-mediated mRNA degradation pathways (Fig. 7C). Enrichment within such pathways predicts proestrus-specific differences in global and specific mRNA translation^56^ relative to males. Conversely, diestrus-selective sex differences were also apparent within estrogen receptor signaling and sirtuin signaling and pathways, whose activity levels were both predicted to be lower in diestrus females than in males (Fig. 7D). The former may be expected, given the lower measured levels of estradiol in the diestrus phase relative to proestrus rats^57^—who did not differ from males in estrogen receptor signaling proteins or phosphoproteins. Diestrus-specific enrichment in sirtuin signaling—involved in metabolism and cellular homeostasis^58^—is consistent with the ontological enrichment of energy metabolism-related proteins and phosphoproteins in diestrus compared to male rats. Moreover, the SIRT1 activator resveratrol has demonstrated effectiveness in alleviating ovariectomy-induced depressive-and anxiety-like behavior in mice via enhanced Sirt1 expression^59^, implicating sirtuin signaling in estradiol-modulated behavioral outcomes. Interestingly, separate IPA analyses of phosphoproteins whose levels significantly differed between either proestrus females and males (Fig. 7E) or diestrus females and males (Fig. 7F) resulted in high overlap among the top 10 enriched signaling pathways between these comparisons. Enrichment of differentially-abundant phosphoproteins from both comparisons yielded estrous-cycle independent positive Z-Scores predicting greater female-specific activation within PKA signaling, synaptogenesis signaling and TCA cycle II pathways, consistent with the grouped female phosphoprotein-selective pathway analysis presented in Fig. 3C.

Taking a broadened look at the data presented up to this point, the large scale of sex-dependent differences in both the dHPC proteome and phosphoproteome can be partially explained by estrous cycle-dependent differences observed within females between proestrus and diestrus rats. Indeed, apart from the comparison between proestrus and diestrus female rats, direct comparisons between diestrus females and males (Fig. 6B) as well as proestrus females and males (Fig. 6A) clearly demonstrate that the majority of significant proteome-level differences between females and males can be attributable primarily to diestrus females, as much more minimal dHPC differences were observed in total protein between proestrus female versus male rats. Interestingly, the magnitude and direction (greater in female vs. male) of difference observed in differentially-abundant phosphoproteins was similar between diestrus and proestrus females when either was compared to males (Fig. 6).

Supported by the findings presented in Sect. “Widespread effects of estrous cycle on the female dHPC proteome”, these data collectively suggest that the hormonal fluctuations throughout the estrous cycle may have a greater impact on total protein abundance than on transient changes in protein phosphorylation levels. Similar widespread effects of the estrous cycle on mRNA expression levels have been observed within the mPFC of diestrus and proestrus female rats when compared to males^55^. While performed at the transcript level, results from this work are consistent with the present findings at the protein level supporting extensive reorganization of synaptic function across the estrous cycle compared to males, in addition to predominantly diestrus-specific sex differences in regulation of energy and metabolic processes. While such large-scale proteomics studies assessing sex and estrous cycle differences within the dHPC are lacking, some work has been performed within the HPC as a whole. One such study conducted in mice surprisingly detected no significant sex differences in the hippocampal proteome^45^. However, the entire dorsal-to-ventral HPC axis was used here, and female data were not stratified by estrous cycle^45^. Given the findings presented herein, it is likely that the lack of subregion-specific differentiation across estrous cycle stages within female mice may have blunted the observation of any potential sex differences that may have existed. It is worth noting that species differences may also help explain differences between studies, as mice—while also exhibiting normal 4–5 day cycles—present hormonal fluctuation patterns for estradiol and progesterone distinct from that observed in rats^34,57,60^. Indeed, in support of the present work, proteomic investigation of the rat HPC—while not subregion-specific—has identified sex- and estrous cycle-dependent differences in the abundance of proteins involved in synaptic function^53^, structural/cytoskeletal organization^53^, as well as protein synthesis and degradation machinery^52^. Collectively, the findings presented here add to existing literature supporting sex- and estrous cycle-dependent protein expression within cognitive and emotion-relevant brain regions, and expand this work by providing the first large-scale parallel analysis of sex and estrous cycle effects on the dHPC proteome and phosphoproteome in male and female rats.

### Rapid sex-specific changes in the dHPC phosphoproteome following low-dose ketamine

As previously indicated, the involvement of the dHPC in both cognitive and emotion-relevant functions make this region an interesting convergence point for investigation of potential sex differences at baseline, as well as sex-specific response to fast-acting antidepressant medications that may affect outcomes in both of these behavioral arenas. To this end, the dHPC has been implicated in ketamine’s rapid antidepressant-like actions in rodents^25,26^ and in humans^61^, and long-term ketamine-induced changes within this region have been associated with its sex-specific pro-hedonic effects^27^. Unfortunately, the mechanisms underlying the heightened female behavioral sensitivity appear distinct from those established in males^26^, and are as of yet unclear. Therefore, having delineated baseline contributions of both sex and estrous cycle to differences in dHPC proteome and phosphoproteome of male and female rats, we next sought to build upon that knowledge to determine whether such baseline differences may predict rapid changes in signaling pathway activation following a low dose (2.5 mg/kg) ketamine which is behaviorally effective in female—but not male—rats.

**Figure 8:**
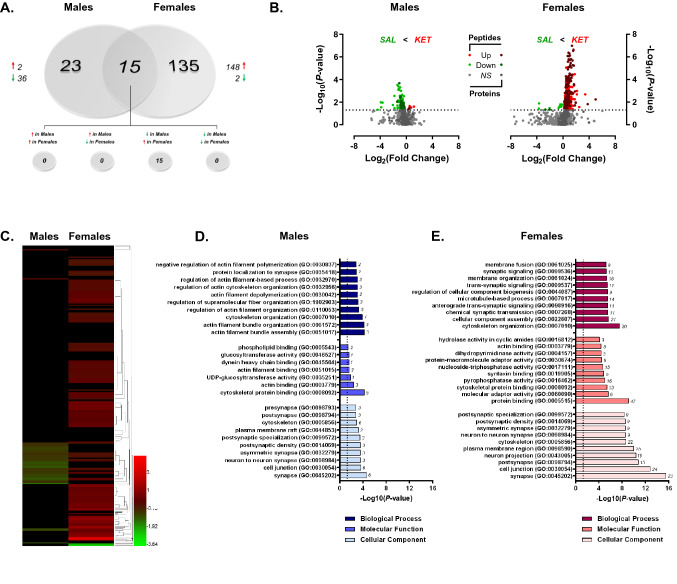
Rapid sex-specific changes in the dHPC phosphoproteome following low-dose ketamine. (**A**) Venn diagram depicting the number of phosphoproteins with significant differential abundance between ketamine (KET)- and saline (SAL)-treated male and female rats. Red arrows indicate greater levels observed in KET- *vs.* SAL-treated rats. (**B**) Volcano plots showing significant KET-induced alterations in phosphoprotein abundance in male (left) and female (right) rats. Log_2_(Fold Change) values > 0 (red) indicate higher—and those < 0 (green) lower—abundance in KET-treated rats compared to SAL groups. Points above the horizontal dashed line represent proteins with *p* < 0.05 (FDR threshold set at 1%). (**C**) Heat map illustrating fold change of phosphoproteins whose abundance was significantly affected by KET in male and female rats. Top 10 most significant (*p* < 0.05) gene ontology (GO) enrichment terms per category of phosphoproteins with significant KET-induced differential abundance in the male (**D**) and female (**E**) rat dHPC. Heat map (**C**) was produced in GENE-E (Broad Institute; https://software.broadinstitute.org/GENE-E/).

Paralleling the heightened behavioral sensitivity of female rats to this low dose of ketamine, acute administration at 2.5 mg/kg induced rapid increases in protein phosphorylation selectively in female rats relative to saline-treated female controls (Fig. 8A–C). For this comparison, 1362 peptides were confidently identified and quantified from TiO2-enriched dHPC lysate, 1076 for which phosphosites could be determined. Of these peptides, 382 unique phosphoproteins were submitted for label-free quantitation (Supplementary Tables S21 and S22). In ketamine-treated females, 150 phosphoproteins were significantly differentially abundant compared to saline-treated controls, 98.7% of whose levels were increased following treatment (Fig. 8A). When comparing ketamine-treated males to saline controls, 1029 peptides representing 235 unique phosphoproteins were identified and quantified (Supplementary tables S23 and S24)—of those, levels of only 38 phosphoproteins were found to be significantly altered by ketamine treatment. Thirty-six of those differentially abundant proteins exhibited lower levels in treated vs. control rats (Fig. 8A). Among those phosphoproteins whose levels were significantly affected by ketamine treatment in both sexes (15), all were increased in females and decreased in males. Visual representations of this bi-directional regulation of protein phosphorylation are clearly depicted in the volcano plots in Fig. 8B, as well as the heat map in Fig. 8C which shows well the opposite regulation of commonly-affected phosphoproteins in response to ketamine treatment.

Ontology analyses of differentially abundant phosphoproteins altered by ketamine in males (Fig. 8D) and females (Fig. 8E) reveal surprising similarities in that both sexes show strong enrichment of proteins predominantly in synapse-associated cellular locations. However, the similarities in ketamine-induced changes between male and female rats appear to be limited to cellular compartment. Whereas the few male phosphoproteins significantly affected by ketamine are primarily involved in actin binding and cytoskeletal organization, female-specific phosphoprotein changes are strongly enriched in both synaptic signaling processes as well as structural protein dynamics localized to the synapse (Fig. 8E). Of course, the low number of differentially-abundant phosphoproteins in ketamine-treated males limits the ability to compare statistical overrepresentation analyses of GO terms.

**Figure 9:**
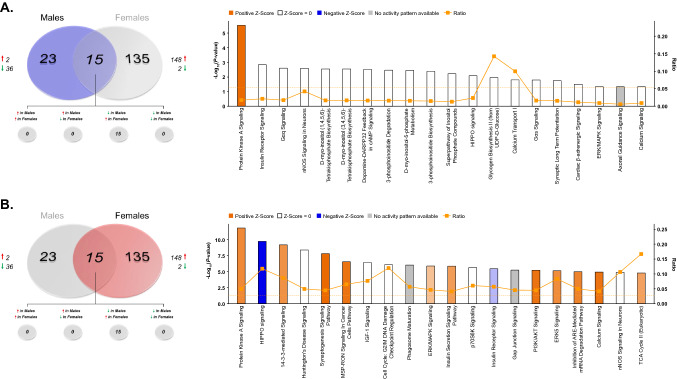
IPA enrichment analyses of ketamine-induced changes in protein phosphorylation in the dHPC of male and female rats. (**A**,**B**) Canonical pathway enrichment for phosphoproteins significantly differentially abundant in ketamine *vs.* saline-treated male (**A**) and female (**B**) rats 30 min following treatment. Based upon the direction of fold change of significant differentially abundant phosphoproteins within the dataset, the *Z*-Score serves as a predictor of pathway activation states by assessing the match between the observed and predicted (evidence-based) up/down regulation patterns^41^. Pathways are colored by predicted activation state: orange (activated) or blue (inhibited), where darker colors represent higher absolute *Z*-scores. Z-scores for pathways colored in gray could not be calculated due to insufficient evidence for a prediction of either activation or inhibition to be made. The ratio (solid orange line) represents the number of molecules within the comparison dataset meeting the cutoff criterion (*p* < 0.05; Fisher’s Exact Test), divided by the number of molecules comprising a given pathway within the reference set (Ingenuity Knowledge Base). Bar graphs were produced in Ingenuity Pathway Analysis (IPA) software (QIAGEN, Inc.).

Given the lack of knowledge regarding signaling mechanisms mediating the enhanced behavioral sensitivity of female rats to low-dose ketamine compared to males^26,62^, phosphoproteins differentially-abundant between ketamine- and saline-treated female rats were submitted to IPA for canonical pathway enrichment analysis in order to compare ketamine-induced signaling pathway activation between sexes. This analysis yielded a surprising parallel in the pathway exhibiting the greatest statistical enrichment of ketamine-affected phosphoproteins in both males (Fig. 9A; Supplemental Table S25) and females (Fig. 9B; Supplemental Table S26)—PKA signaling. Of course, the primary difference exhibited between sexes was the direction of ketamine-induced change in phosphoprotein abundance. Whereas ketamine significantly reduced levels of phosphoproteins within this pathway, nearly all of those observed in females were greatly elevated when compared to saline-treated controls. Synaptogenesis signaling was also selectively enriched in ketamine-treated females; however, this is not surprising given the large number of shared proteins (e.g. CAMK2A, CAMK2G, PRKAR2A, GSK3B) between this pathway and PKA signaling whose abundance was preferentially increased by low-dose ketamine in female, but not male, rats relative to their saline-treated counterparts.

### Ketamine-induced changes in the dHPC phosphoproteome of proestrus and diestrus female rats

**Figure 10:**
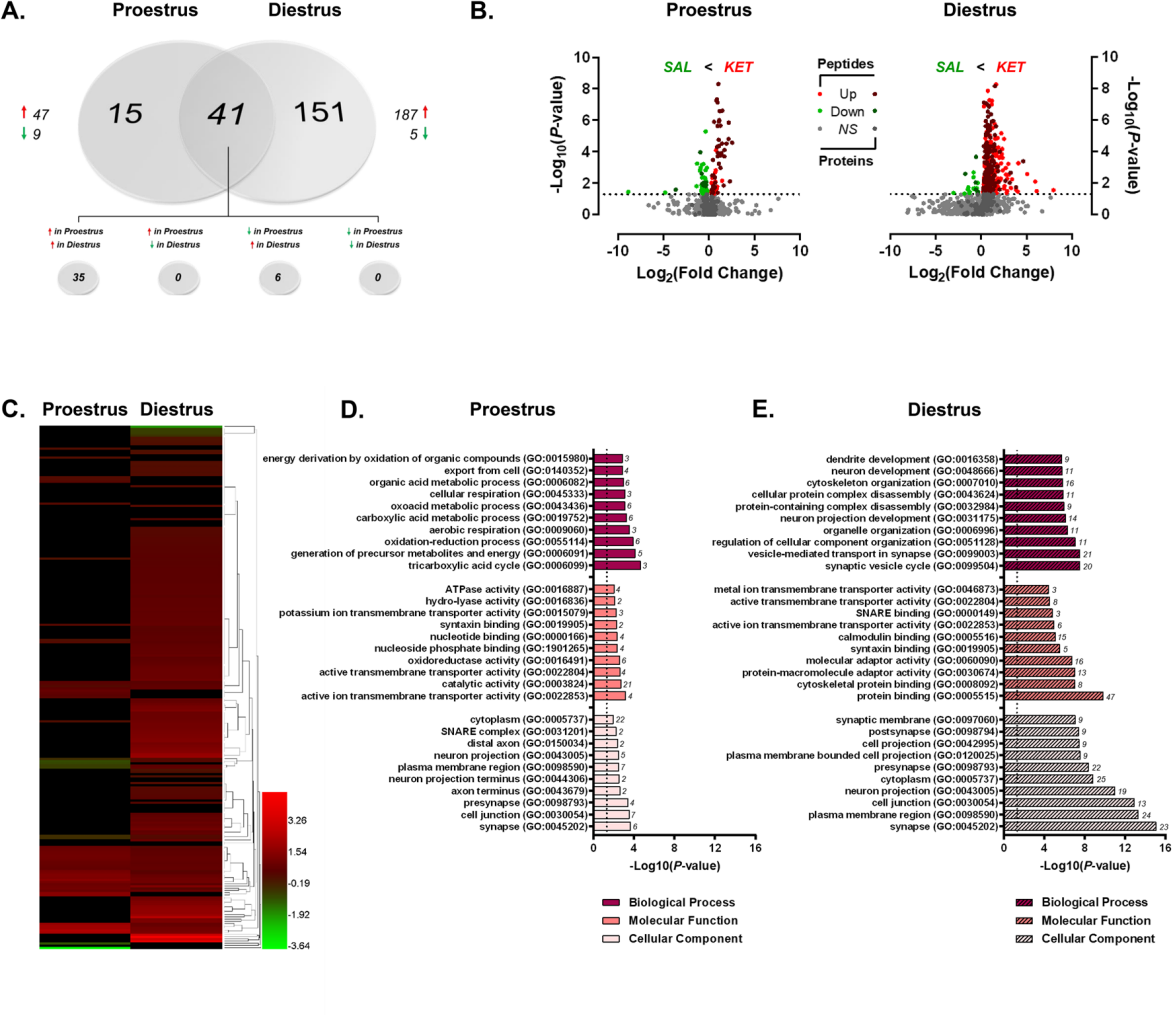
Ketamine-induced changes in the dHPC phosphoproteome of proestrus and diestrus female rats. (**A**) Venn diagram depicting the number of phosphoproteins with significant estrous cycle-dependent differential abundance between ketamine (KET)- and saline (SAL)-treated proestrus or diestrus female rats. Red arrows indicate greater levels observed in KET- *vs.* SAL-treated rats. (**B**) Volcano plots showing significant KET-induced alterations in dHPC phosphoprotein abundance in proestrus (left) and diestrus (right) female rats. Log_2_(Fold Change) values > 0 (red) indicate higher—and those < 0 (green) lower—abundance in KET-treated rats compared to SAL groups. Points above the horizontal dashed line represent proteins with *p* < 0.05 (FDR threshold set at 1%). (**C**) Heat map illustrating fold change of phosphoproteins significantly affected by KET in either proestrus or diestrus female rats. Top 10 most significant (*p* < 0.05) gene ontology (GO) enrichment terms per category of phosphoproteins with significant KET-induced differential abundance in proestrus (**D**) and diestrus (**E**) female rats. Heat map (**C**) was produced in GENE-E (Broad Institute; https://software.broadinstitute.org/GENE-E/).

Finally, with the data presented herein demonstrating variable estrous cycle-dependent dHPC protein and phosphoprotein abundance in female rats, as well as the hormone dependence of heightened female behavioral sensitivity to low-dose ketamine^26,27^, it is plausible that ketamine may exert cycle stage-dependent changes within this brain region. Therefore, we analyzed ketamine-induced alterations in the dHPC phosphoproteome independently in proestrus and diestrus female rats to determine whether circulating hormone levels impacted the phosphoprotein profile observed in ketamine-treated female rats compared to controls. While the magnitude of changes was greater in diestrus than in proestrus female rats treated with ketamine (vs. saline-treated females), the direction of change was similar between both groups of females (Fig. 10A). Of the 998 peptides and 251 unique dHPC phosphoproteins quantified in ketamine- vs. saline-treated proestrus females (Supplementary Tables S27 and S28), 56 significantly differentially-abundant phosphoproteins were identified—47 (83.9%) of those phosphoproteins were significantly increased by ketamine treatment (Fig. 10A). Further, 1669 phosphopeptides mapping to 353 unique phosphoproteins were confidently identified and quantified in ketamine-treated diestrus female rats. Ketamine significantly increased the abundance of 187/192 (97.4%) phosphoproteins exhibiting differential abundance in ketamine-treated diestrus females when compared with controls (Fig. 10A; Supplementary Tables S29 and S30). The similar effect on the increasing trend of ketamine-induced phosphoprotein abundance changes observed between proestrus and diestrus females is evident in the volcano plots pictured in Fig. 10B, shown by the greater number and magnitude of positive relative to negative log_2_(fold changes) in both groups of females. In addition, as depicted in the heat map in Fig. 10C (as well as in the overlapping portion of the Venn diagram; Fig. 10A), a majority (35/41) of the commonly-regulated phosphoproteins between proestrus and diestrus females presented greater abundance in ketamine- vs. saline-treated female rats.

Interestingly, while differentially-abundant phosphoproteins affected by ketamine treatment were enriched in synapse and cell projection (i.e. axonal) cellular compartments in both ketamine-treated proestrus (Fig. 10D) and diestrus (Fig. 10E) females, the GO biological processes and molecular functions overrepresented in each group diverged slightly based on estrous cycle stage. For example, ketamine treatment in proestrus females affected levels of phosphoproteins primarily related to energy derivation, cellular respiration and ATPase activity (Fig. 10D); whereas those altered by drug treatment in diestrus females were more-so related to dendritic/neuronal development and cytoskeletal organization, in addition to transport and binding activity at the synaptic membrane (e.g. SNARE, syntaxin and calmodulin binding).

**Figure 11:**
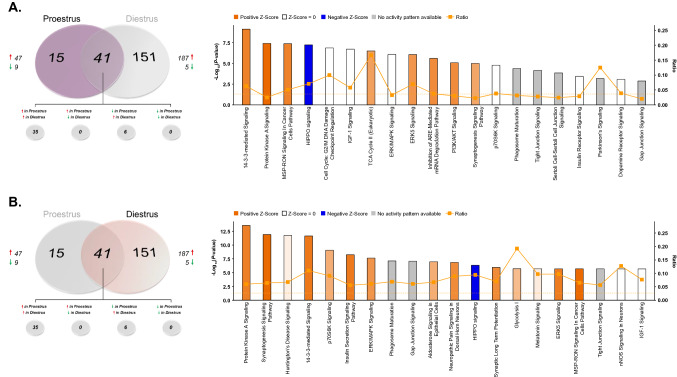
Estrous cycle-specific IPA enrichment analyses of differential phosphoprotein abundance following ketamine treatment in the female rat dHPC. Canonical pathway enrichment of phosphoproteins whose abundance was significantly altered by low-dose ketamine in proestrus (**A**) or diestrus (**B**) female rats. Based upon the direction of fold change of significant differentially abundant phosphoproteins within the dataset, the *Z*-Score serves as a predictor of pathway activation states by assessing the match between the observed and predicted (evidence-based) up/down regulation patterns^41^. Pathways are colored by predicted activation state: orange (activated) or blue (inhibited), where darker colors represent higher absolute *Z*-scores. Z-scores for pathways colored in gray could not be calculated due to insufficient evidence for a prediction of either activation or inhibition to be made. The ratio (solid orange line) represents the number of molecules within the comparison dataset meeting the cutoff criterion (*p* < 0.05; Fisher’s Exact Test), divided by the number of molecules comprising a given pathway within the reference set (Ingenuity Knowledge Base). Bar graphs were produced in Ingenuity Pathway Analysis (IPA) software (QIAGEN, Inc.).

To further aid the interpretation of these estrous cycle-dependent phosphoprotein changes affected by ketamine, IPA enrichment was performed separately on significantly differentially-abundant phosphoproteins in ketamine-treated proestrus (Fig. 11A; Supplementary Table S31) and diestrus (Fig. 11B; Supplementary Table S32) females relative to their saline-treated counterparts. Of particular interest, the PKA signaling pathway is among the most significantly enriched in both proestrus and diestrus females. Consistent with the ontological overrepresentation analyses, ketamine treatment also affected dHPC phosphoprotein abundance in proestrus female rats within the TCA cycle II pathway, involved in derivation of energy from organic molecules for cell growth and division. Conversely, diestrus females exhibited enrichment in differentially-abundant phosphoproteins involved in synaptogenesis signaling and synaptic long-term potentiation—consistent with ontological enrichment in SNARE/syntaxin binding and vesicle-mediated transport at the synapse.

### Sex-dependent activation of PKA signaling by low-dose ketamine

**Figure 12:**
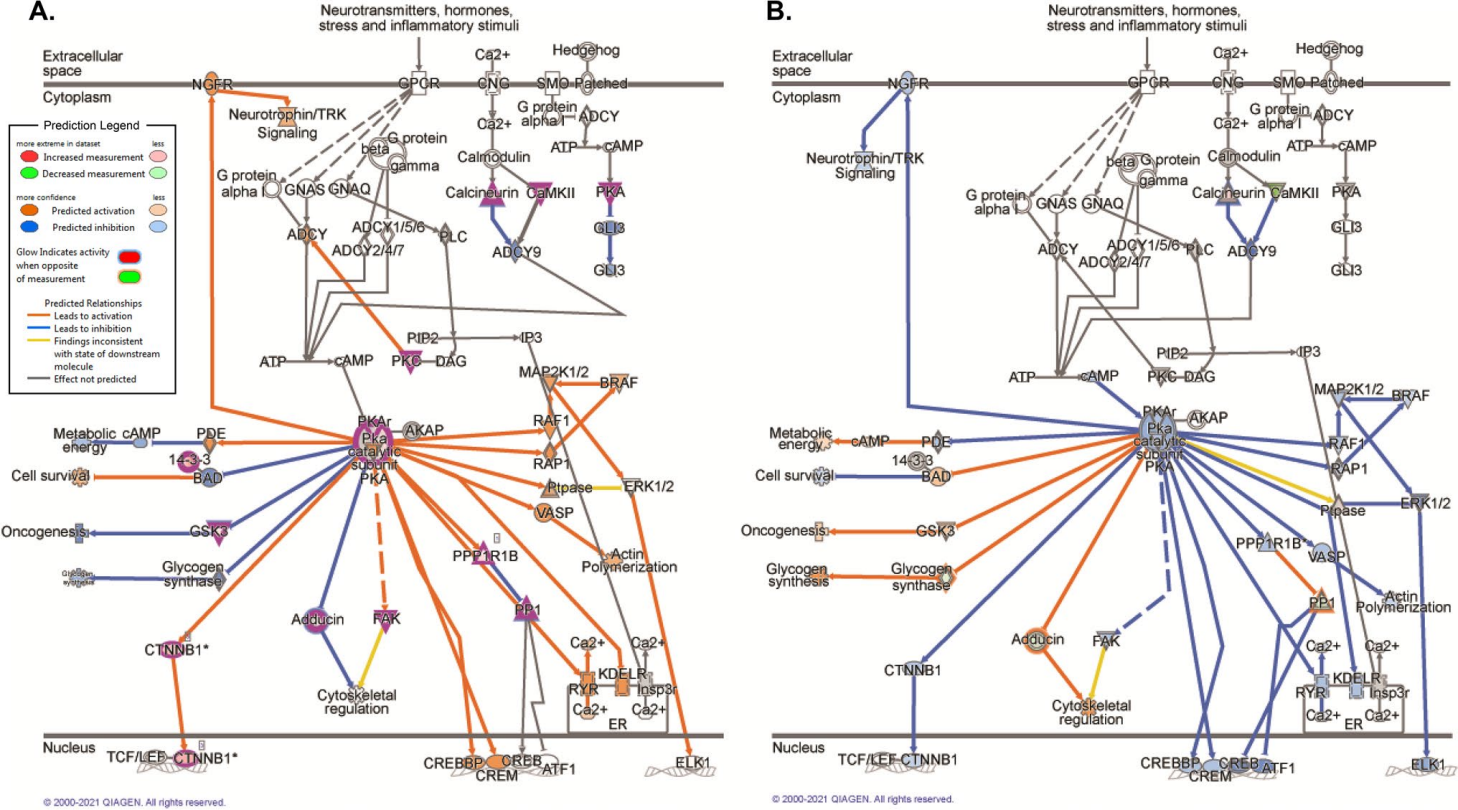
Rapid sex-selective induction of PKA signaling by low-dose ketamine in the rat dHPC. Depiction of phosphoprotein abundance significantly altered by ketamine in female (**A**) and male (**B**) rats within the PKA signaling pathway. Phosphoproteins meeting the significance criteria (*p* < 0.05; FDR cutoff = 1%) in each dataset are outlined in fuchsia. IPA’s Molecule Activity Predictor (MAP) was superimposed upon the current dataset, and used fold change values (ketamine *vs.* saline groups) of differentially-abundant phosphoproteins (*p* < 0.05) within the pathway to predict the impact of such changes on upstream and downstream PKA signaling activity. Pathway images were produced in Ingenuity Pathway Analysis (IPA) software (QIAGEN, Inc.).

**Figure 13:**
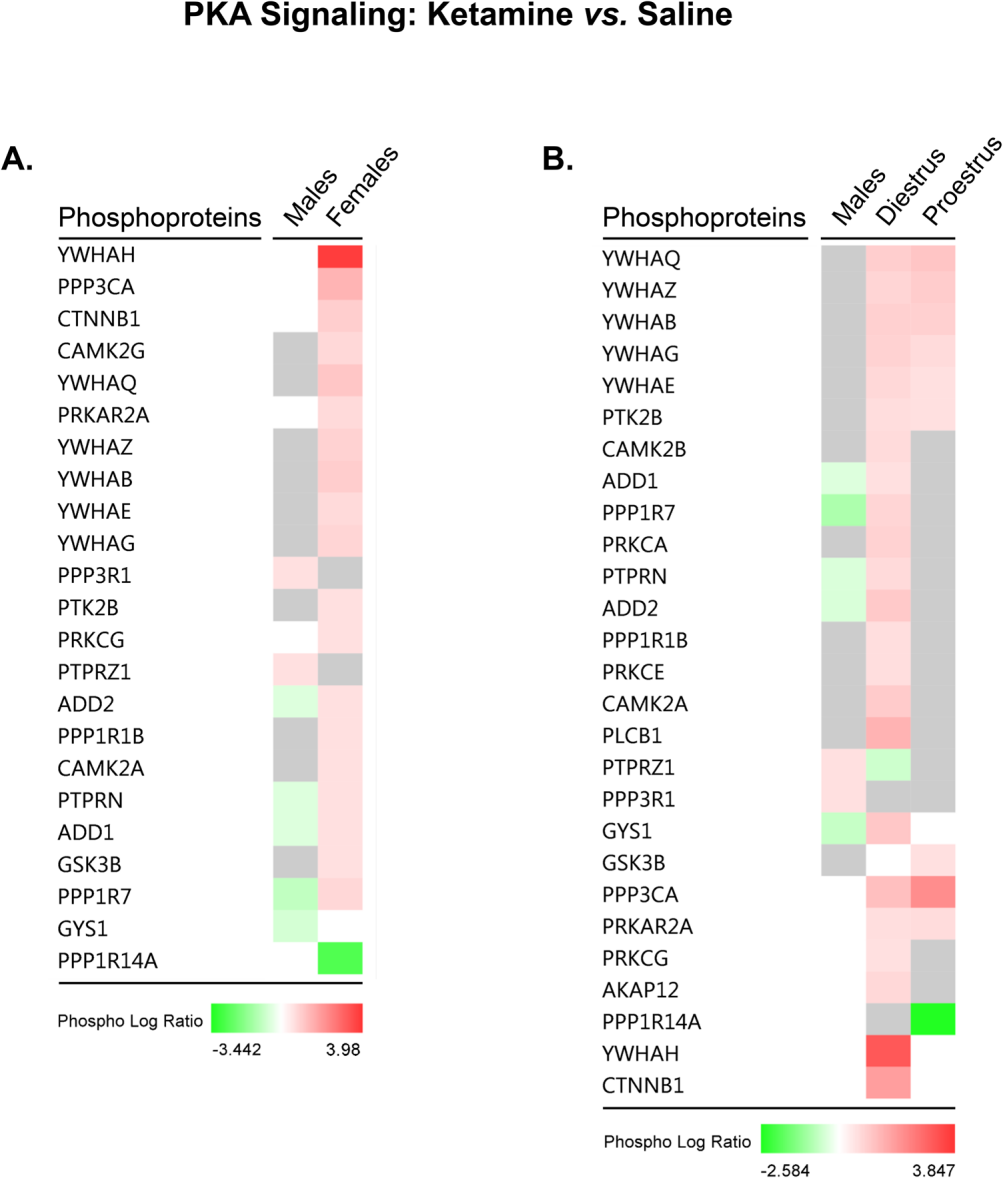
Sex- and estrous cycle-dependent modification of PKA signaling phosphoproteins by ketamine within the dHPC. Heat maps comparing the log fold change (“Phospho Log Ratio”) by sex (**A**) and estrous cycle (**B**) of dHPC phosphoproteins whose abundance was significantly altered by 2.5 mg/kg ketamine in intact male and female rats. Heat maps were generated using Ingenuity Pathway Analysis (IPA) software (QIAGEN, Inc.).

Taken together, these data suggest that regardless of hormonal status, low-dose ketamine preferentially activates PKA signaling in females, but not males. Figure 12 illustrates the predicted effects of ketamine-induced changes in phosphoprotein abundance on activity of molecules upstream and downstream of cytosolic PKA activation based on the observed data in female (12A) and male (12B) rats, respectively. Sex- (Fig. 13A) and estrous cycle (Fig. 13B)-specific comparisons are also presented for individual phosphoproteins selectively affected by ketamine within the PKA signaling pathway. Here, it is interesting to note that while ketamine induced similar changes in phosphorylation of several proteins common to both proestrus and diestrus female rats, a number of non-overlapping phosphoproteins were also affected by ketamine in an estrous cycle-dependent manner. Though the net predicted effect of these changes was increased activation of PKA signaling in both proestrus and diestrus females, the functional significance of these estrous cycle-specific differences remains unclear. It should be noted that significant differences in PKA signaling exist at baseline between proestrus and diestrus females and may contribute to some of the cycle-dependent effects of low-dose ketamine within this pathway.

In both proestrus and diestrus females, ketamine results in significantly increased levels of phosphorylated KAP2 (or PRKAR2A), or the type II-alpha regulatory subunit of PKA, selectively in female rats—this change is persistent across estrous cycle stage and is therefore likely to be sex-dependent rather than hormonally-modulated. Increased PKA phosphorylation of this regulatory subunit is a cAMP-dependent event. Based on the present dataset, the greater abundance of either phosphorylated PKCγ or CAMKIIα observed in ketamine-treated females relative to controls could result in elevated cAMP levels via activation of adenylyl cyclase. Indeed, recent work has demonstrated rapid (within 20 min) regulation of CAMKIIα phosphorylation in the hippocampus by low-dose ketamine (5 mg/kg) in male mice critical for behavioral response to this dose, as well as delayed induction of GluA1 24 h after treatment^63^. As well, consistent with the observed intermediary decrease in phosphorylation of a regulatory PP1 subunit (PPP1R7) in ketamine-treated females, increased PKA activation has been shown to lead to transient insertion of calcium-permeable AMPARs, and contribute to hippocampal LTP in a CAMKII-dependent manner^64^. Predicted downstream effects based on these female-specific actions of low-dose ketamine within the dHPC are presented in Fig. 12, and include predicted downstream activation of neurotrophin signaling, as well as CREB-mediated transcription. CREB-mediated transcription of BDNF could be one mechanism consistent with our previous work demonstrating elevated BDNF protein levels selectively in the dHPC of treatment-responsive female rats 24 h following administration of the same low dose of ketamine used herein^27^. While it remains unclear to what extent these changes may contribute to sex- and estrous cycle-dependent cognitive and depression-relevant behavioral sensitivity to low-dose ketamine, future work should aim to elucidate potential functional relevance in both stress and non stress-exposed animals.

Of particular relevance to the present work, MRI imaging of rats following a single injection of 10 mg/kg ketamine revealed short-term reductions in activation of the anterodorsal hippocampus, which the authors proposed may confer transient pro-cognitive effects of ketamine^65^. While repeated treatment with low-dose ketamine has been shown to impair dHPC-relevant working memory in a dose- and dosing-frequency-dependent manner^66^, investigations examining acute effects of behaviorally-relevant doses of ketamine on hippocampal-dependent cognitive functions (e.g. spatial object recognition) in male and female rats are absent, and potential estrous cycle effects are unknown. Nonetheless, given the relevance of the dHPC to spatial memory, it is possible that the observed female-specific increase in PKA-mediated signaling may contribute to ketamine’s effects within this cognitive domain in a sex-dependent manner. Activation of PKA signaling by ketamine mediated via elevated cAMP levels is known to impact cognitive processes^67^, and spatial memory is affected by elevated dHPC cAMP levels in a biphasic manner^17^. More specifically, Lyman and colleagues (2021) recently demonstrated that acute increases in dCA1 pyramidal neurons in chronically-stressed male and female mice impaired object location memory, and blunted response to the antidepressant citalopram in this behavioral task^17^. Conversely, chronically-elevated dHPC cAMP levels improved performance in the same object location memory task. These effects were determined to be mediated via alterations in HCN channel trafficking and surface expression and altered excitability of dCA1 pyramidal neurons. Given that cAMP-mediated regulation of PKA activity modulates CA1 pyramidal neuron excitability^68^, it is possible that the female-specific activation of PKA signaling by ketamine observed in this study may impact spatial recognition task performance in a sex-dependent manner. Indeed, recent work has shown that PKA is required for LTP in the dHPC of female, but not male, Sprague Dawley rats, suggesting some sex-specificity in the role this pathway may play in this hippocampal subregion^69^.

## Conclusions

Taken together, the present work provides the first analysis of global differences in the rat dHPC proteome and phosphoproteome across sex and estrous cycle. Within this region, baseline proteomic and phosphoproteomic differences between male and female rats were generally lower for differentially abundant total proteins, but higher for phosphoproteins in female compared to male rats as a whole. Here, these proteins were predominantly related to PKA signaling, synaptogenesis/synaptic long-term potentiation pathways and mitochondrial function. Ontological analysis revealed that the major sex-dependent differences in the dHPC proteome and phosphoproteome globally affect structural protein dynamics, mitochondrial energy metabolism level of the synapse and mitochondria. Of note, synapse-associated processes were also determined to be hormone-dependent when female rats in proestrus were compared to those in diestrus; however, the most notable cycle-dependent differences in the dHPC proteome and phosphoproteome were those enriched in mitochondrial compartments and affecting mitochondrial function and energy metabolism, in general. Significant enrichment in PKA signaling-related phosphoproteins was also apparent upon independent comparison of TiO2-enriched dHPC fractions from proestrus versus diestrus females, where higher abundance predicted greater activation of this pathway in normal diestrus female rats.

Interestingly, a low dose of ketamine that is behaviorally effective in female, but not male, rats induces rapid activation of PKA signaling selectively in female rats regardless of hormonal status—as evidenced by greater abundance of phosphoproteins within this pathway in ketamine- versus saline-treated diestrus and proestrus female rats (Figs. 12, 13B). Female-specific phosphoproteins significantly regulated by ketamine were enriched in synaptic signal transduction biological processes localized, perhaps unsurprisingly, to synaptic cellular compartments. Conversely, lower PKA-related phosphoprotein levels also were identified in the male rat dHPC 30 min following ketamine administration. Taken together, these novel findings identify PKA signaling as a potential sex-specific mechanism triggered in the dHPC by low-dose ketamine selectively in female rats. Given the presence of baseline sex- and estrous cycle-dependent differences in phosphoprotein levels within this pathway, the present work also emphasizes the importance of considering whether baseline differences (if any) may predict or impact drug-induced signaling changes relevant to the behavioral endpoint of interest, in general. While assessment of the involvement of PKA signaling in the enhanced female behavioral sensitivity to ketamine is beyond the scope of this study, future work addressing the potential functional relevance of this signaling cascade to cognitive and/or depressive-like behavioral effects of low-dose ketamine would be pertinent.

Although few studies exist examining the effect of estrous cycle on the behavioral efficacy of low-dose ketamine, previous work from our lab found greater antidepressant-like effects of ketamine in proestrus compared to diestrus female mice^28^. However, no specific investigation of estrous cycle-dependent antidepressant-like or cognitive effects of low-dose ketamine exists for female rats. This is an important distinction given the differences in patterns of hormonal fluctuation across the estrous cycle between mice and rats^34,60^, such that cycle-specific behavioral effects of ketamine in mice may not translate to rats. Given the present findings of modest estrous cycle-specific effects of ketamine on dHPC protein phosphorylation patterns, such an examination of the effects of estrous cycle on ketamine’s behavioral efficacy in dHPC-mediated behaviors (e.g., spatial object recognition) warrants further investigation. With the known absence of effects of estrous cycle on peripheral and dHPC levels of ketamine in female rats^31^, it is more likely that potential ketamine-induced behavioral differences between proestrus and diestrus rats would be due to pharmacodynamic rather than pharmacokinetic differences (as identified herein). In addition, whether or not similar ketamine-induced changes in dHPC protein phosphorylation in females occur at behaviorally-relevant doses (as well as higher, anesthetic doses) in males is unknown and should be addressed in future investigations. Indeed, understanding phosphorylation changes following ketamine at doses associated with different behavioral outcomes may help to clarify their potential functional importance in both sexes. Finally, provided the evidence of sex-specific effects of low-dose ketamine on antidepressant-like efficacy and protein expression within the ventral hippocampus of female mice^28^, future phosphoproteomic analyses within the ventral hippocampus may help to clarify whether similar sex-specific changes in phosphorylation patterns exist for this subregion.

## Supplementary Information

Supplementary Table S1.

Supplementary Table S2.

Supplementary Table S3.

Supplementary Table S4.

Supplementary Table S5.

Supplementary Table S6.

Supplementary Table S7.

Supplementary Table S8.

Supplementary Table S9.

Supplementary Table S10.

Supplementary Table S11.

Supplementary Table S12.

Supplementary Table S13.

Supplementary Table S14.

Supplementary Table S15.

Supplementary Table S16.

Supplementary Table S17.

Supplementary Table S18.

Supplementary Table S19.

Supplementary Table S20.

Supplementary Table S21.

Supplementary Table S22.

Supplementary Table S23.

Supplementary Table S24.

Supplementary Table S25.

Supplementary Table S26.

Supplementary Table S27.

Supplementary Table S28.

Supplementary Table S29.

Supplementary Table S30.

Supplementary Table S31.

Supplementary Table S32.
